# Registered Nurse Workload and Resident Outcomes in Nursing Homes: A Scoping Review of Workforce Sustainability and Nursing Management

**DOI:** 10.1155/jonm/6082303

**Published:** 2026-10-13

**Authors:** Joseph Almazan, Assem Zhakupova, Zhanas Baimagambet, Nuraiym Nurmukhambetova, James Montegrico, Srinivasa Bolla, Cris S. Adolfo

**Affiliations:** ^1^ Department of Medicine School of Medicine Nazarbayev University Kerey and Zhanibek Khans St 5/1 Astana 010000 Kazakhstan nu.edu.kz; ^2^ Department of Biomedical Sciences School of Medicine Nazarbayev University Kerey and Zhanibek Khans St 5/1 Astana 010000 Kazakhstan nu.edu.kz; ^3^ School of Nursing University of North Carolina at Charlotte Charlotte North Carolina 28223 USA uncc.edu; ^4^ Registered Nurse St. André Health Care Biddeford Maine USA

**Keywords:** nursing homes, registered nurses, resident outcomes, staffing, workload

## Abstract

**Aim:**

To map RN workload, staffing indicators, resident outcomes, workforce sustainability and nursing‐management context in nursing homes.

**Background:**

Staffing ratios and hours per resident day (HPRD) quantify staffing but not role complexity, interruptions, supervisory demands, resident acuity, continuity or work environment.

**Evaluation:**

Following JBI guidance and PRISMA‐ScR, five databases were searched using an RN‐specific strategy and updated on 4 September 2026. Following source‐level eligibility reassessment and reconciliation of the updated search, 20 studies meeting the RN‐specific resident‐outcome criteria formed the primary synthesis. Seven studies that did not meet these criteria but provided relevant workforce or nursing‐management evidence were retained as a separately labelled contextual evidence layer and excluded from the primary synthesis and PRISMA included‐study count. Primary studies underwent full data charting, whereas contextual studies underwent restricted extraction for interpretive purposes only.

**Key Issues:**

Evidence was predominantly observational. Greater RN availability and more favourable work environments were generally associated with better resident outcomes, although findings varied by outcome, timing, staffing configuration and organisational context.

**Conclusions:**

RN workload is multidimensional and cannot be reduced to staffing ratios alone. Current evidence does not establish a universal RN staffing threshold or support causal conclusions across nursing‐home settings.

**Implications for Nursing Management:**

Managers should interpret staffing metrics alongside resident acuity, staffing continuity, workload intensity, skill mix, agency use and work‐environment indicators.

## 1. Introduction

Global population ageing has intensified the demand for high‐quality, person‐centred long‐term care (LTC) as the number of older people and the complexity of their care needs continue to increase [1]. In nursing homes, registered nurses (RNs) play an important role in maintaining resident safety and quality of care through clinical assessment, monitoring, coordination and oversight of care [2]. Their work extends beyond direct resident care and is shaped by responsibilities for care coordination, supervision, documentation and responding to changing resident needs [2, 3]. RN staffing is therefore important not only in terms of the amount of nursing time available but also in how nursing resources are organised and translated into everyday care [4–6].

Staffing measures such as nurse‐to‐resident ratios and hours per resident day (HPRD) are commonly used to quantify nursing resources in nursing homes [7–9]. However, numerical staffing measures do not fully capture the broader demands associated with nursing work. These may include resident acuity, interruptions and time pressure, supervisory responsibilities, work organisation, emotional or ethical strain and staffing instability [3, 10, 11]. Consequently, similar staffing levels may operate differently depending on skill mix, supervisory coverage, staffing configuration, team functioning and the broader work environment [4–6, 12].

A substantial body of literature has examined the relationship between nursing‐home staffing and resident outcomes. Previous syntheses have already established this area of research. Jutkowitz et al. [9] systematically reviewed evidence concerning nurse staffing and resident outcomes in nursing homes, while Blatter et al. [13] synthesised systematic reviews examining staffing and resident safety. These reviews indicate that higher RN staffing and a more favourable skill mix are often associated with better resident outcomes, although findings vary according to the outcome assessed, staffing measure, study design and organisational context. The contribution of the present review therefore does not rest on an absence of previous staffing–outcome syntheses. Rather, it extends this literature by examining RN workload as a broader organisational construct that encompasses staffing quantity alongside workload intensity, work organisation, workforce sustainability and management context.

Evidence concerning these broader dimensions remained dispersed across studies that used different definitions, measures and outcomes [9, 13]. Differences in staffing models, resident acuity, workforce stability, organisational characteristics and resource contexts further complicated interpretation and limited the direct transferability of findings across nursing‐home settings [6, 14, 15]. Given this conceptual and methodological heterogeneity, we used a scoping approach to map the breadth of the evidence, clarify how researchers had conceptualised and measured RN workload and identify areas in which the evidence remained underdeveloped [16]. This scoping review aimed to synthesise and map the available evidence on RN workload, RN‐specific staffing indicators, resident outcomes, workforce sustainability and the nursing‐management context in nursing homes. We also sought to identify gaps in the literature and provide direction for future research, workforce planning, policy development and evidence‐informed nursing management.

## 2. Background

Nursing homes are an increasingly important component of health and LTC systems as populations age and the prevalence of multimorbidity, frailty, cognitive impairment and complex care needs increases. According to the WHO [1], the global population aged 60 years and older is projected to reach 2.1 billion by 2050. This demographic change is accompanied by growing demand for LTC services capable of responding to increasingly complex health and support needs [2].

As life expectancy increases and chronic conditions become more prevalent, nursing homes are expected to provide far more than basic assistance with daily living. Residents may require ongoing clinical monitoring, management of comorbidities, preventive care, medication management, psychosocial support and timely responses to changes in health status. Within these settings, RNs contribute clinical expertise, surveillance, coordination and continuity of care. Their relational and clinical roles are therefore important to maintaining the safety, dignity and well‐being of nursing‐home residents [6].

Despite this central role, nursing homes continued to face challenges in achieving adequate RN staffing while maintaining manageable workloads. In this review, we defined RN workload as the range of demands placed on RNs in nursing‐home settings, including direct resident care, clinical assessment and judgement, care coordination, documentation, supervisory responsibilities, interruptions, cognitive demands and emotional labour. High workload could arise from excessive assignments, fragmented workflows, frequent interruptions, limited recovery time and the complexity of residents’ care needs [3, 10]. These pressures could reduce the time and attention available for individualised care and were associated with workforce concerns such as job dissatisfaction, burnout and reduced workforce sustainability [4, 10].

At the same time, resident care outcomes encompassed a broad range of indicators relating to health, safety, service use, psychosocial wellbeing and quality of life [5, 9]. These included falls, pressure injuries, infections, hospitalisations, emergency‐department use, medication‐related outcomes, functional decline and quality of life [5, 7, 17, 18]. The evidence suggested that favourable resident outcomes were associated not only with staffing levels, but also with workload intensity, skill mix, resident acuity, staffing continuity, team functioning and organisational support [4–6, 12].

Empirical studies frequently reported associations between RN staffing and resident outcomes in nursing homes. Higher RN staffing was associated with favourable outcomes, including reduced hospital or emergency‐department utilisation and improvements in selected nursing‐sensitive outcomes [7, 14, 19, 20]. However, these associations varied across outcomes, settings, staffing configurations and temporal patterns [6, 15]. Staffing quantity represented only one dimension of RN workload. Measures such as HPRD quantified available RN staffing but did not fully capture resident acuity, staffing continuity, supervisory coverage, agency use, workflow demands or the broader organisational context in which care was delivered [4, 5, 12, 18]. Nursing homes with similar RN staffing levels could therefore experience substantially different workload conditions and capacities to respond to residents’ needs.

The consequences of RN workload also extended to the nursing workforce. Unfavourable work environments were associated with greater RN burnout and job dissatisfaction, raising concerns about workforce sustainability and continuity of care [4, 5]. From a management perspective, staffing ratios alone therefore provided an incomplete indication of whether RNs had sufficient capacity to deliver safe, responsive and person‐centred care.

Organisational context further shaped how available nursing resources were translated into care. Skill mix, supervisory coverage, staffing continuity, agency use, team functioning and the work environment could influence both experienced workload and resident outcomes [4–6, 12, 18]. These contextual differences may help explain why associations between RN staffing and resident outcomes varied across nursing homes. Previous systematic reviews had already established an important evidence base on nursing‐home staffing, skill mix and resident safety and quality outcomes [9, 13]. However, these syntheses focused primarily on staffing levels and staffing configurations rather than RN workload as a broader construct encompassing work organisation, workload intensity, workforce sustainability and organisational context. We therefore examined RN‐specific staffing indicators together with these broader dimensions.

Given the heterogeneity of concepts, measures, methods and settings, we used a scoping review to map the available evidence, clarify how researchers had conceptualised and measured RN workload and identify areas in which the evidence remained underdeveloped [16]. Rather than estimating a single staffing effect or proposing a universal staffing threshold, we sought to distinguish staffing quantity from the broader construct of RN workload and examine how both related to resident outcomes across organisational contexts. The synthesis was intended to inform future research, workforce planning, policy development and evidence‐informed nursing management without extending conclusions beyond the strength of the available evidence.

### 2.1. Objectives

• To map RN‐specific evidence on the associations between workload, staffing‐related workload indicators and resident outcomes in nursing homes.
• To distinguish numerical staffing measures from broader dimensions of RN workload, work organisation and workforce sustainability.
• To identify evidence‐informed considerations for nursing management while clearly distinguishing observed associations from causal effects or established intervention effects.

## 3. Methods

This scoping review followed the Joanna Briggs Institute (JBI) methodology for scoping reviews and was informed by the framework originally proposed by Arksey and O’Malley [16, 21]. The review process comprised identification of the research question, identification of relevant studies, study selection, data charting, synthesis and reporting of findings and consultation [16, 21]. Reporting followed the Preferred Reporting Items for Systematic Reviews and Meta‐Analyses extension for Scoping Reviews (PRISMA‐ScR) [22], while PRISMA 2020 guidance was used to structure the study‐selection flow and document the updated search process [23]. The protocol, including the review objectives and methodological approach, is publicly available on the Open Science Framework (OSF): https://doi.org/10.17605/OSF.IO/BMXFT.

### 3.1. Stage 1: Identifying the Research Question

The review was guided by the following questions:

• What RN‐specific evidence is available on the association between workload or staffing‐related workload indicators and resident outcomes in nursing homes?
• How do the work environment, workforce sustainability, skill mix, staffing continuity and management context shape or contextualise these associations?

RN workload was defined as the range of demands placed on RNs in nursing‐home practice, including direct resident care, clinical assessment, care coordination, supervision, documentation, interruptions, cognitive demands, emotional labour and time pressure. To maintain an explicitly RN‐focused evidence base, studies were eligible only when RN‐specific exposures or findings could be distinguished from those of other staff categories. Findings reported only for broad nursing‐staff groups, licensed nurses, nurse aides, nurse practitioners or mixed personnel were not treated as RN workload evidence unless the RN component was separately reported and linked to an eligible resident outcome.

Resident outcomes were defined as measurable indicators of health, safety, service use, medication‐related quality, psychosocial wellbeing or quality of life among nursing‐home residents. Evidence relating to workforce and organisational factors was handled in two analytically distinct layers. Studies reporting a separable RN‐specific exposure together with an eligible resident outcome contributed to the 20‐study primary synthesis. Seven studies from the original review that did not meet the primary RN‐specific resident‐outcome criterion were retained as clearly labelled contextual sources because they informed workload conceptualisation, workforce sustainability, staffing instability, management context or advanced nursing roles and models. These studies were not used to infer RN‐specific effects on resident outcomes.

### 3.2. Stage 2: Identifying Relevant Studies

#### 3.2.1. Search Strategy and Data Sources

The original review employed a systematic search across five electronic databases. During peer review, an eligible RN‐specific study that had not been captured by the original strategy was identified, indicating that greater search sensitivity was required. In response, the eligibility of the existing evidence base was re‐audited at source level, and the search strategy was revised to improve retrieval of RN‐specific studies.

The original search included PubMed, ScienceDirect, Scopus, Web of Science and CINAHL and covered English‐language, peer‐reviewed studies published from 1 January 2000 to 15 February 2025. Search terms represented four broad concepts: nursing‐home setting, nursing staff, workload or staffing and resident outcomes. During revision, the search strategy was broadened after an eligible RN‐specific study not captured by the original search highlighted insufficient search sensitivity. The revised strategy centred on three core concepts: nursing‐home or residential long‐term‐care setting, RN and workload or staffing. Resident outcomes were assessed during eligibility screening rather than required as a mandatory search block. The five databases were rerun on 4 September 2026 using the revised strategy.

The revised core search concepts were as follows: (“registered nurse∗” OR RN OR “professional nurse∗”) AND (workload OR staffing OR “staffing level∗” OR “hours per resident day” OR HPRD OR “skill mix” OR “work environment” OR “staffing stability” OR “agency staff∗”) AND (“nursing home∗” OR “long‐term care facilit∗” OR “care home∗” OR “residential aged care”)

Database‐specific subject headings, field tags, truncation, syntax, date restrictions and full search strategies are reported in Supporting Table 3. The five databases were rerun on 4 September 2026 using the revised RN‐specific strategy. No duplicate records were identified in the update‐search set (*n* = 0). Final eligibility decisions were reconciled with the source‐level re‐audit, resulting in 20 unique studies in the RN‐specific primary synthesis.

#### 3.2.2. Inclusion and Exclusion Criteria

Eligibility was re‐audited using the Population, Concept and Context framework and the criteria presented in Table 1. Particular attention was given to the separability of RN‐specific evidence. For inclusion in the primary synthesis, an RN‐specific workload or staffing exposure had to be separately identifiable, and at least one eligible resident outcome had to be reported. Studies that did not meet this criterion were not used to support RN‐specific associations with resident outcomes. Seven studies from the original review were retained in a separate contextual evidence layer because they addressed management practice, licensed‐nurse work organisation, mixed‐staff shortages or instability, RN workforce well‐being, nurse‐practitioner models or mixed‐nurse workload. This approach retained management‐relevant contextual evidence while keeping it analytically separate from the RN‐specific resident‐outcome synthesis.

**TABLE 1:** Inclusion and exclusion criteria.

Criterion	Inclusion	Exclusion
Population	Studies conducted in nursing homes or equivalent long‐term residential care settings in which RN‐specific workload, staffing, work‐environment, skill‐mix or continuity data could be extracted separately.	Studies in which RN findings could not be separated from those of certified nursing assistants, nursing assistants, LPNs/LVNs, care aides, nurse practitioners or mixed staff categories.
Concept	Studies reporting an RN‐specific workload or staffing‐related exposure together with at least one resident health, safety, service‐use, medication‐related, psychosocial or quality‐of‐life outcome.	Workforce‐only studies without a resident outcome, or studies of staffing or workload in which a separable RN exposure or finding was not reported.
Context	Nursing homes, care homes with nursing, or equivalent long‐term residential care settings.	Acute hospitals, home care, community services, rehabilitation settings, or mixed settings in which nursing‐home data could not be separated.
Publication year	Peer‐reviewed studies published from 1 January 2000 through 4 September 2026.	Studies published outside the eligible period.
Study types	Quantitative, qualitative and mixed‐methods primary research with sufficient full‐text information to assess RN‐specific eligibility.	Reviews, editorials, commentaries, protocols, abstracts, conference‐only reports, theses or dissertations and nonpeer‐reviewed reports. Such sources could inform the background but were not included in the primary‐study synthesis.
Language	English‐language publications.	Non‐English‐language publications.

### 3.3. Stage 3: Selecting Studies

#### 3.3.1. Screening Process

The original search identified 510 records: CINAHL (*n* = 20), PubMed (*n* = 85), Scopus (*n* = 22), Web of Science (*n* = 358) and ScienceDirect (*n* = 25). After 30 duplicates were removed, 480 records underwent title and abstract screening. Fourteen were excluded at this stage, leaving 466 reports for full‐text assessment. All were retrieved and assessed for eligibility. Of these, 450 were excluded and 16 studies were included in the original review.

During revision, the 16 originally included studies were reassessed using the more explicit RN‐specific resident‐outcome criteria. Nine met the criteria for the RN‐specific primary synthesis. Seven studies, Anderson et al. [24], Bowers et al. [3], Chen et al. [25], Munkeby et al. [10], Sinha et al. [26], Tchouaket et al. [27] and Väisänen et al. [11], did not provide a sufficiently separable RN‐specific resident‐outcome relationship and were retained as contextual evidence. These studies are reported separately in Supporting Table 2 and were used only to inform management, workforce, work‐organisation, staffing‐instability and professional‐role context. They were not used to support RN‐specific associations with resident outcomes.

The sensitivity re‐audit identified eight additional RN‐specific primary studies. Re‐examination of 32 extended‐retrieval full‐text records identified four studies that met the primary eligibility criterion: Alexander [28], Cho et al. [29], Konetzka et al. [14] and Mukamel et al. [15]. Konetzka et al. [14] had already been included and was therefore not counted again. Alexander [28], Cho et al. [29] and Mukamel et al. [15] were consequently added as three newly recovered unique studies. In total, 9 retained studies, 8 additional studies identified through the sensitivity re‐audit and 3 newly recovered studies comprised the final RN‐specific primary synthesis of 20 unique studies. The seven contextual studies remained analytically separate.

The five prespecified databases, PubMed, ScienceDirect, Scopus, Web of Science Core Collection and CINAHL (EBSCOhost), were rerun on 4 September 2026 using the revised RN‐specific strategy reported in Supporting Table 3. No duplicate records were identified in the update‐search set (*n* = 0). Following update screening and source reconciliation, the final RN‐specific primary evidence set comprised 20 unique studies. The seven contextual studies were reported separately and were not included in this primary total. The complete study‐selection and evidence‐classification process is presented in Figure 1.

**FIGURE 1:**
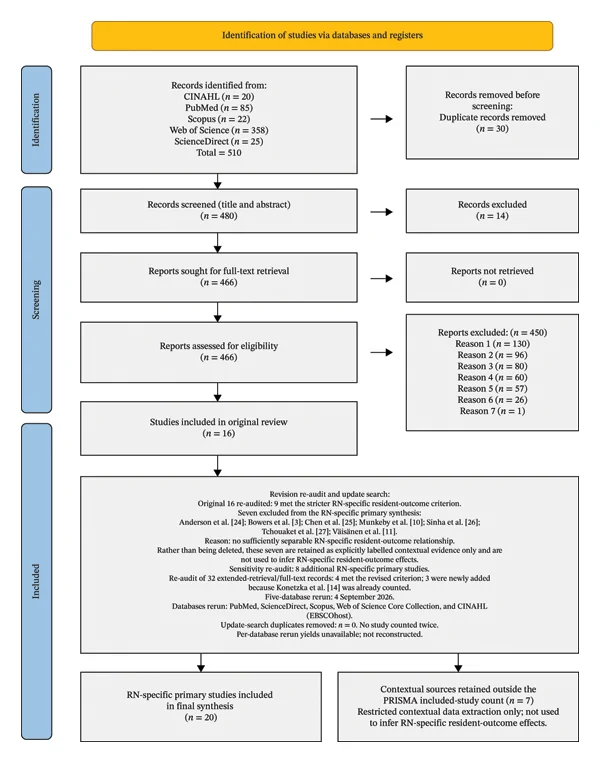
PRISMA flow diagram illustrating the selection process for the RN‐specific primary synthesis. Reason 1: review or systematic review articles. Reason 2: studies did not address resident care outcomes. Reason 3: not conducted in nursing home settings. Reason 4: did not address the relationship between RN workload and resident care outcomes. Reason 5: included nonregistered nursing personnel. Reason 6: insufficient data. Reason 7: outside eligible publication date window (January 1, 2000–February 15, 2025). Note: Original record‐level counts are retained as historical search counts. The update search was rerun on 4 September 2026; update‐search duplicates removed = 0. The final RN‐specific primary synthesis contains 20 unique studies; the seven named studies above are retained only as contextual evidence. Four of the 32 re‐audited records met the revised criterion; only three were newly added because Konetzka et al. [14] was already included. Per‐database rerun yields were unavailable and were not reconstructed.

#### 3.3.2. Data Management and Analysis Tools

Zotero was used for reference management and deduplication. Screening decisions, reasons for exclusion and changes to data charting were documented in the review audit trail. Citation metrics were not used to determine study eligibility or influence the synthesis (see Figure 2).

**FIGURE 2:**
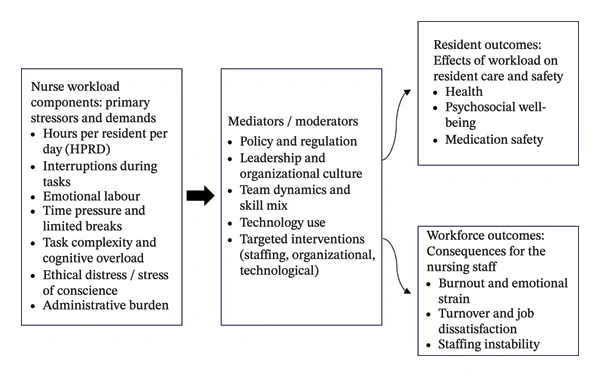
Conceptual model of RN workload influences on care and workforce outcomes. Note: No third‐party logos or reproduced image assets are used in Figure 2.

### 3.4. Stage 4: Charting the Data

Data charting used a piloted two‐layer extraction form. For the 20 studies meeting the RN‐specific primary criterion, Assem Zhakupova, Nuraiym Nurmukhambetova and Zhanas Baimagambet extracted author and year, country, study design, setting and sample, RN‐specific workload or staffing exposure, resident outcome, analytical approach, effect estimate or qualitative finding and relevant workforce or organisational context. Joseph Almazan and James Montegrico verified each entry against the original source.

For the seven studies retained as contextual evidence, data extraction was restricted to citation details, study context, the reason for exclusion from the primary RN‐specific synthesis, the relevant contextual domain and the source‐supported finding used in the interpretation. Contextual domains included management practice, work organisation, staffing instability, workforce well‐being, advanced nursing models and mixed‐nurse workload. These studies were not included in the RN‐specific primary synthesis, study counts, effect summaries or statements concerning RN‐specific associations with resident outcomes. Disagreements regarding data extraction or evidence classification were resolved through discussion and re‐examination of the original source.

### 3.5. Stage 5: Collating, Summarising and Reporting the Results

We conducted a descriptive and interpretive synthesis rather than a meta‐analysis and organised the evidence into two distinct layers. The 20 RN‐specific primary studies formed the main synthesis and were grouped into domains reflecting the review questions: RN staffing intensity and resident outcomes; work environment and workforce sustainability; contextual and nonlinear associations; and implications for nursing management and policy. The seven contextual studies were synthesised separately and used to inform interpretation of management, workforce, work‐organisation, staffing‐instability and professional‐role issues. They were not counted as primary studies and were not used to support RN‐specific associations with resident outcomes. Numerical estimates were reported only when traceable to the original source, and observational associations were not interpreted as evidence of intervention effectiveness.

### 3.6. Stage 6: Consultation Exercise

In this final stage, a consultation exercise was conducted to enhance the credibility, interpretability and practical relevance of the synthesis. Four experts with experience in nursing home care, nursing workload, gerontological nursing and workforce or quality‐improvement research were invited because of their subject expertise and familiarity with LTC practice. The experts reviewed the preliminary themes, data‐charting summaries, conceptual model and interpretation of the findings. Their feedback focused on clarifying the distinction between RN workload, staffing levels, skill mix, resident acuity and organisational context; improving the terminology used for resident outcomes; and ensuring that management implications were relevant to nursing home administrators and directors of nursing. Their comments led to refinements in theme labels, clearer reporting of the RN‐focused scope, more cautious wording around causal inference, and a strengthened discussion of staffing policy and workload monitoring. The consultation did not alter the number of included studies or the eligibility decisions. After these refinements, the experts reviewed the final outputs to confirm that the synthesis was coherent and aligned with nursing workload and resident care outcome evidence.

## 4. Results

### 4.1. Study Selection and Characteristics

Following reconciliation of the source‐level re‐audit and the five‐database update search conducted on 4 September 2026, 20 unique studies met the criteria for the RN‐specific primary synthesis. Nine came from the original 16‐study evidence set, eight were additional RN‐specific studies identified through the sensitivity re‐audit, and three were newly recovered from the 32 previously extended‐retrieval full‐text records: Alexander [28], Cho et al. [29] and Mukamel et al. [15]. Konetzka et al. [14] was one of four studies in the 32‐record re‐audit that met the criterion but had already been included and was therefore not counted again. Seven studies from the original review, Anderson et al. [24], Bowers et al. [3], Chen et al. [25], Munkeby et al. [10], Sinha et al. [26], Tchouaket et al. [27] and Väisänen et al. [11], were retained as contextual sources. They are reported separately because they inform workforce, management, work organisation, staffing instability, or professional‐role context but do not provide sufficiently separable RN‐specific resident‐outcome evidence.

### 4.2. Data Extraction and Evidence Classification

Full data were charted for the 20 RN‐specific primary studies and are reported in Table 2, Part A. The seven studies retained as contextual evidence were charted using a restricted extraction framework and are reported in Table 2, Part B and Supporting Table 2. For these seven studies, only source‐supported contextual information relevant to management practice, work organisation, staffing instability, workforce well‐being, nurse‐practitioner models or mixed‐nurse workload was retained. Their findings were not used to support RN‐specific associations with resident outcomes and did not contribute to the primary synthesis. Accordingly, the PRISMA included‐study count and the RN‐specific primary synthesis both remained at *n* = 20, while the seven contextual studies were reported separately.

**TABLE 2:** Data extraction and evidence classification in the revised review: part A, RN‐specific primary synthesis (*n* = 20); part B, contextual evidence retained after re‐audit (*n* = 7).

Author(s) & year	Country	Study design	Sample size and setting	RN‐specific exposure/contextual domain	Findings
Abdulai [20]	United States	Retrospective facility‐level analysis	14,860 Medicare‐ and Medicaid‐certified long‐term care facilities	RN HPRD; linear regression adjusted for facility characteristics	Higher RN HPRD was associated with lower excess outpatient emergency‐department use for long‐ and short‐stay residents.
Alexander [28]	United States (Missouri)	Retrospective cross‐sectional facility‐level analysis	510 Missouri nursing homes; Nursing Home Compare data; 14 quality measures	RN HPRD analysed separately from LPN and CNA HPRD; ANOVA/contrasts	RN staffing levels showed significant differences for ADL decline and short‐stay pressure‐ulcer measures, while RN‐specific effects were not significant for continence or short‐stay pain. RN HPRD was analysed separately from LPN and CNA HPRD.
Blatter et al. [12]	Switzerland	Retrospective cohort/time‐to‐event	937 residents; 1138 admissions across seven units of a 180‐bed multisite nursing home	RN supervisory coverage and RN/CNA skill mix; time‐to‐event modelling	Pressure‐injury risk varied with RN supervisory coverage and staffing configuration, supporting a contextual rather than staffing‐only interpretation.
Chappell et al. [30]	United States	Cross‐sectional facility‐level analysis	10,436 long‐term care facilities	RN staffing hours, with licensed, LPN and nursing‐aide staffing examined separately; linear regression	Each unit increase in RN staffing hours was associated with a 2.25% decrease in inappropriate antipsychotic prescribing.
Charlwood et al. [6]	England	Retrospective longitudinal observational study	320 care homes with nursing across two large providers	RN staffing/skill mix and temporary RN use; longitudinal/nonlinear models	Associations were nonlinear: both very low and very high RN proportions could be associated with higher risk depending on outcome and context.
Cho et al. [29]	South Korea	Secondary analysis of national administrative data	5679 nursing‐home residents in National Health Insurance Service data	Number of RNs; mixed‐effect linear and multinomial logistic models	Greater numbers of RNs were associated with lower resident mortality, while overall nursing‐home quality ratings increased as RN numbers increased.
Cho et al. [5]	South Korea	Cross‐sectional observational	117 RNs and 513 residents in 39 nursing homes	RN staffing levels and work environment; GEE	More RNs and a more favourable work environment were associated with better resident quality of life; RN job dissatisfaction, burnout and turnover intention were also reported. https://doi.org/10.1111/jocn.16689
Choi et al. [8]	South Korea	Cross‐sectional	216 nurses in 62 nursing homes	Number of residents per RN per shift, work environment and RN education; logistic regression	More residents per RN per shift were associated with higher odds of RN‐reported medication errors, pressure ulcers and urinary tract infections. A poor work environment was associated with higher odds of several adverse events.
Harrington et al. [31]	United States (California)	Cross‐sectional facility‐level analysis	California nursing homes in state and public quality datasets	RN HPRD/staffing ratings	Facilities with RN staffing below the recommended minimum had a higher probability of reported COVID‐19 resident infections.
Khoja et al. [18]	United States	Cross‐sectional	11,183 nursing homes; 571,106 long‐stay residents (from 1,115,304 Medicare FFS enrolees)	Discipline‐specific staffing and agency use; regression modelling	The association between staffing and injurious falls depended on the surrounding staffing configuration, arguing against a simple single‐discipline linear model.
Konetzka et al. [14]	United States	Longitudinal; fixed effects and instrumental variables	399,206 resident assessments from 1366 freestanding nursing homes in five states	RN HPRD and RN skill mix; fixed effects and instrumental‐variable methods	Greater RN staffing was associated with lower pressure‐sore and urinary‐tract‐infection incidence; methods addressed staffing endogeneity.
Lee et al. [32]	South Korea	Secondary analysis of national nursing‐home data	3389 nursing homes	Proportion of RNs in the nursing workforce; district‐level OLS fixed‐effects regression using nursing‐home data aggregated across 249 administrative districts	A higher RN proportion was significantly associated with lower COVID‐19 infection measures; associations with mortality were lower in direction but did not reach statistical significance.
Mukamel et al. [15]	United States	Retrospective national facility‐day analysis	15,718 nursing homes reporting Payroll Based Journal data, 2017–2019	Daily RN, LPN and CNA hours entered separately; facility fixed‐effect Poisson models	Higher prior‐day RN staffing was associated with fewer subsequent hospital/ED transfers, while higher same‐day staffing was associated with more transfers.
Park & Min [2]	South Korea	Qualitative descriptive	10 RNs from six nursing homes	RN perspectives on systemic and work‐organisation factors; thematic analysis	RNs identified insufficient RN staffing, poor working conditions, unclear RN roles, absence of a formal nursing‐director position and lack of a transition‐care system as systemic factors affecting perceived care quality and safety.
Pradhan et al. [33]	United States	Cross‐sectional structural equation model	12,900 free‐standing nursing homes	RN HPRD within a structure‐process‐outcome model	Neighbourhood social deprivation was associated with staffing patterns and quality pathways.
Shin et al. [7]	South Korea	Longitudinal survey	74 nursing homes	RN HPRD; longitudinal multilevel modelling	An additional unit of RN HPRD was associated with about a 10.5% decrease in deteriorated quality‐of‐care outcomes.
Shin et al. [34]	South Korea	Cross‐sectional	51 nursing homes	RN HPRD; regularised negative‐binomial regression	Higher RN HPRD was related to fewer hospitalisations due to infection or disease.
White et al. [4]	United States	Cross‐sectional linked survey/administrative data	674 RNs in 245 nursing homes	RN work environment; multivariable analysis	Better work environments were associated with fewer pressure ulcers and hospitalisations and with markedly lower RN burnout and job dissatisfaction.
Yang et al. [19]	United States	Retrospective facility‐level secondary analysis	14,325 Medicare/Medicaid‐certified nursing homes	RN, LPN and CNA HPRD staffing patterns; cluster analysis and multivariate generalised estimating equations	Nursing homes in the high‐RN staffing cluster had significantly lower rehospitalisation and emergency‐department use than homes in the high‐LPN cluster; the high‐CNA versus high‐LPN comparison showed a similar but nonsignificant trend.
Yoon et al. [17]	United States	Cross‐sectional secondary data analysis	14,548 Medicare‐ or Medicaid‐certified facilities	RN HPRD and RN skill mix; generalised linear mixed models	Greater RN HPRD and RN skill mix were associated with lower odds of inappropriate psychotropic use deficiency citations.

*Part B. Contextual evidence retained after RN-specific re-audit (n = 7). These studies were excluded from the RN-specific primary synthesis and from the PRISMA included-study count. Contextual data charting was restricted to study context/domain, the reason for primary ineligibility and source-supported findings used for interpretation; no RN-specific resident-outcome effect estimate from these studies was included in the primary synthesis.*					
Anderson et al. [24]	United States	Quantitative; cross‐sectional correlational field study	164 nursing homes; staff RNs included	Management practice and organisational context	Retained as contextual evidence: management practices were related to resident outcomes, but RN workload was not isolated as a separable RN‐specific resident‐outcome exposure.
Bowers et al. [3]	United States	Qualitative; grounded dimensional analysis	18 nurses from two long‐term‐care facilities	Licensed‐nurse time pressure and work organisation	Retained as contextual evidence: the study provides rich evidence on time pressure and work organisation, but RN and LPN findings are not sufficiently separable for the RN‐specific primary synthesis.
Chen et al. [25]	United States	Longitudinal facility‐level analysis	8466 nursing homes	Staff shortages, staffing levels and resident outcomes	Retained as contextual evidence: staffing shortages and resident outcomes were examined using mixed/overall staffing measures without a sufficiently separable RN‐specific resident‐outcome estimate.
Munkeby et al. [10]	Norway	Cross‐sectional study	2093 long‐term‐care providers, including 950 RNs and 1143 other care providers, working in nursing homes and home‐care services	Stress of conscience and workforce wellbeing	Retained as contextual evidence: the study is RN‐specific but centres on a workforce outcome (stress of conscience) and does not provide the required RN‐specific resident outcome for the primary synthesis.
Sinha et al. [26]	United States	Cross‐sectional administrative analysis	11,840 nursing homes	Staffing instability, turnover and quality	Retained as contextual evidence: instability and turnover were analysed primarily as total/mixed staffing constructs rather than as a separable RN‐specific resident‐outcome effect.
Tchouaket et al. [27]	Canada (Québec)	Economic evaluation/cost‐savings analysis	538 residents across six long‐term‐care facilities	Nurse‐practitioner model and cost outcomes	Retained as contextual evidence: the intervention concerns nurse practitioners and broader staffing strategy rather than RN workload as defined for the primary synthesis.
Väisänen et al. [11]	Finland	Cross‐sectional study	503 nurses from 44 assisted‐living facilities with 24‐h assistance: 76 RNs and 427 practical nurses	Workday characteristics, stress and time pressure	Retained as contextual evidence: workload‐related stress and time pressure were examined in mixed nursing personnel, without a sufficiently separable RN‐specific resident‐outcome relationship.

### 4.3. RN Workload and Staffing‐Related Measures

Across the 20 RN‐specific primary studies, RN workload was most often represented indirectly through structural staffing measures rather than through direct multidimensional workload instruments. Common indicators included RN HPRD, RN proportion or skill mix, residents per nurse, supervisory coverage, temporary or agency staffing and day‐to‐day staffing variation. Shin et al. [7], Yoon et al. [17], Abdulai [20] and Konetzka et al. [14] used RN‐specific staffing measures, whereas Charlwood et al. [6], Blatter et al. [12] and Khoja et al. [18] examined RN staffing within broader staffing configurations. Park and Min [2] identified inadequate staffing, poor working conditions, unclear RN roles and limited formal nursing leadership as systemic factors affecting care quality and safety. Overall, the evidence remained weighted towards staffing quantity as a proxy for workload, with relatively little direct measurement of interruptions, cognitive demand, documentation burden, emotional labour, supervisory span or workload peaks.

### 4.4. Associations Between RN Workload, Staffing and Resident Outcomes

The overall pattern was favourable to greater RN availability, although associations varied by outcome, study design and staffing configuration. Shin et al. [7] found that greater RN HPRD was associated with fewer deteriorated quality‐of‐care outcomes. Choi et al. [8] reported that more residents per nurse were associated with higher incidences of medication errors, pressure ulcers and urinary tract infections. Konetzka et al. [14], using longitudinal data with facility fixed effects and instrumental‐variable methods, found that greater RN staffing was associated with lower incidence of pressure sores and urinary tract infections after accounting for staffing endogeneity. These findings indicate associations between RN staffing intensity and selected resident outcomes but do not establish a universal staffing threshold.

Four studies in the 32‐record re‐audit met the RN‐specific eligibility criterion: Alexander [28], Cho et al. [29], Konetzka et al. [14] and Mukamel et al. [15]. Alexander [28] analysed RN, LPN and CNA HPRD separately and identified staffing‐level differences across continence, activities‐of‐daily‐living decline, pain and pressure‐ulcer quality measures. Cho et al. [29], using data from 5679 nursing‐home residents in South Korea, found that the number of RNs was associated with resident mortality and that overall nursing‐home quality ratings increased as RN numbers increased. Konetzka et al. [14] had already been included in the original evidence set. Mukamel et al. [15], in a national analysis of 15,718 US nursing homes, found that higher prior‐day RN staffing was associated with fewer subsequent hospital and emergency‐department transfers, whereas higher same‐day staffing was associated with more transfers. These findings highlight the importance of RN‐specific measurement, staffing configuration, temporal ordering and possible reverse causation when interpreting staffing‐outcome associations.

Service‐use, infection‐related and medication‐related outcomes also varied across studies. Abdulai [20] found that higher RN HPRD was associated with lower excess outpatient emergency‐department use, while Yang et al. [19] reported associations between RN staffing and skill mix and rehospitalisation and emergency‐department use. Shin et al. [34] found that higher RN HPRD was associated with fewer infection‐ or disease‐related hospitalisations. Harrington et al. [31] and Lee et al. [32] reported associations between RN staffing or RN workforce composition and COVID‐19 outcomes. Yoon et al. [17] found that greater RN HPRD and RN skill mix were associated with lower odds of deficiency citations for inappropriate psychotropic medication use. Chappell et al. [30] reported lower inappropriate antipsychotic use with higher staffing, with RN‐specific interpretation limited to separable RN staffing measures. Cho et al. [5] extended the evidence to resident quality of life, with more RNs and a more favourable work environment associated with higher quality‐of‐life scores.

### 4.5. Work Environment and Workforce Sustainability

The RN‐specific primary evidence indicates that staffing quantity operates within a wider work environment. White et al. [4] found that better nursing‐home work environments were associated with fewer pressure ulcers and hospitalisations and with markedly lower RN burnout and job dissatisfaction. Cho et al. [5] likewise found that a more favourable work environment was associated with better resident quality of life while also documenting RN job dissatisfaction, burnout and turnover intention. Park and Min [2] identified inadequate staffing, poor working conditions, unclear RN roles and lack of formal nursing leadership as systemic factors affecting care quality and safety. Together, these findings support considering workforce sustainability as part of the organisational context of resident care, but they do not establish that burnout or turnover directly cause resident harm.

The seven contextual studies retained from the original review provided additional evidence on management, workforce and work‐organisation factors without contributing to the RN‐specific resident‐outcome synthesis. Anderson et al. [24] contributed evidence on management practice and organisational context; Bowers et al. [3] on licensed‐nurse time pressure and work organisation; Chen et al. [25] and Sinha et al. [26] on mixed‐staff shortages, staffing instability, turnover and quality; Munkeby et al. [10] on RN workforce well‐being; Tchouaket et al. [27] on a nurse‐practitioner model; and Väisänen et al. [11] on stress and time pressure among mixed nursing personnel. These studies were charted separately, and no effect estimate or association from them was used to support the 20‐study RN‐specific resident‐outcome synthesis.

### 4.6. Policy, Staffing Configuration and Organisational Context

Several studies indicated that associations between RN staffing and resident outcomes were conditional rather than strictly linear. Charlwood et al. [6] identified nonlinear associations between RN staffing and skill mix and care quality in English care homes with nursing. Blatter et al. [12] found that pressure‐injury risk varied according to RN supervisory coverage and the surrounding direct‐care staffing configuration. Khoja et al. [18] similarly reported that associations between staffing and injurious falls depended on staffing configuration and agency use. Pradhan et al. [33] found that neighbourhood social deprivation was associated with staffing patterns and quality pathways, highlighting the potential influence of organisational and contextual disadvantage. These findings indicate that RN staffing measures should be interpreted in relation to skill mix, supervisory coverage, agency use and the wider organisational and resource context rather than as isolated indicators.

### 4.7. Evidence‐Informed Nursing Management Considerations and Evidence Gaps

The evidence base did not identify a single tested management intervention or a universal RN staffing threshold. Instead, several management‐relevant dimensions emerged from the primary evidence, including RN availability, skill mix, agency use, supervisory coverage, work environment and workforce sustainability [4–6, 12, 18]. Broader considerations identified across the synthesis included resident acuity, workload intensity and staffing continuity.

Important evidence gaps also remained across the RN‐specific evidence base. Most studies relied on structural staffing indicators, such as RN HPRD, staffing levels, skill mix, or staffing configuration, rather than direct multidimensional measures of experienced RN workload. As a result, factors such as interruptions, cognitive demand, documentation burden, emotional labour, supervisory responsibilities and workload peaks were rarely examined alongside staffing measures. Resident‐reported outcomes were also studied less frequently than administrative safety, clinical or service‐use outcomes and relatively few studies assessed staffing, experienced workload, organisational context and resident outcomes within the same design. These limitations restrict understanding of how staffing levels are translated into day‐to‐day workload and resident outcomes.

### 4.8. Conceptual Framework of the Review Findings

The conceptual framework distinguishes structural staffing capacity, workload demands, experienced RN workload and organisational context. Structural staffing capacity includes RN HPRD, RN skill mix, supervisory coverage, staffing continuity and temporary or agency staffing. Workload demands include resident acuity, task density and changing care requirements, while experienced RN workload encompasses interruptions, documentation demands, cognitive and emotional demands, supervisory responsibilities and time pressure. Organisational factors, including leadership, team organisation, work environment and local resource conditions, may influence how staffing capacity and workload demands relate to resident and workforce outcomes. The framework is intentionally noncausal. It organises the relationships and workload dimensions identified across the review while recognising that several dimensions of experienced workload were not directly or consistently measured in the included studies.

## 5. Discussion

This scoping review maps a 20‐study RN‐specific primary evidence base linking staffing‐related workload indicators, broader work conditions, resident outcomes and workforce sustainability in nursing homes, while retaining seven studies from the original review as a clearly separated contextual evidence layer. Previous syntheses have already examined nursing‐home staffing and resident outcomes [9, 13]. This review distinguishes numerical staffing measures from the broader construct of RN workload and clarifies how contextual evidence can inform interpretation without being treated as separable RN‐specific resident‐outcome evidence.

The source‐level re‐audit provided additional detail to this interpretation because four records within the 32‐record re‐audit met the RN‐specific eligibility criterion: Alexander [28], Cho et al. [29], Konetzka et al. [14] and Mukamel et al. [15]. Alexander [28] analysed RN, LPN and CNA HPRD separately and identified staffing‐level differences across continence, activities‐of‐daily‐living decline, pain and pressure‐ulcer quality measures. Cho et al. [29] added South Korean evidence showing associations between the number of RNs, resident mortality and overall nursing‐home quality ratings. Konetzka et al. [14], already present in the original evidence set, used facility fixed effects and instrumental‐variable methods to address staffing endogeneity and found that greater RN staffing was associated with lower pressure‐sore and urinary‐tract‐infection incidence. Mukamel et al. [15] added a temporal perspective, with higher prior‐day RN staffing associated with fewer subsequent hospital and emergency‐department transfers, while higher same‐day RN staffing was associated with more transfers. All four studies therefore informed the re‐audit interpretation, although only Alexander [28], Cho et al. [29] and Mukamel et al. [15] were newly recovered unique studies because Konetzka et al. [14] had already been included.

Across the 20‐study primary evidence base, greater RN availability or more favourable work environments were generally associated with better resident outcomes, although the pattern was not uniform. Associations were reported for clinical deterioration, pressure injuries and infections, hospital and emergency‐department use, medication‐related quality, COVID‐19 outcomes and resident quality of life [4, 5, 7, 14, 17, 19, 20, 31, 32]. Differences in outcome definitions, staffing measures, analytical adjustment, temporal scale and health‐system context, however, limit direct comparison across studies and preclude interpretation as a single pooled effect. The evidence therefore indicates that RN staffing and the nursing work environment are repeatedly associated with resident outcomes, but it does not support the conclusion that a single staffing level will produce equivalent benefits across nursing homes.

Mukamel et al. [15] is particularly informative because it underscores the importance of temporal ordering when interpreting associations between RN staffing and resident outcomes. Higher RN staffing on the preceding day was associated with fewer subsequent hospital and emergency‐department transfers, whereas higher staffing on the day of transfer was associated with increased transfer rates. This contrasting pattern raises the possibility of reverse causation: staffing levels may increase in response to emerging clinical deterioration or greater care demands on days when transfer becomes necessary. However, given the observational design, this explanation remains plausible rather than established.

Alexander [28] similarly demonstrated that staffing–outcome relationships were neither uniform nor consistent across resident quality indicators when RN, LPN and CNA HPRD were examined separately. Together, these findings reinforce the nonlinear and configuration‐dependent associations reported by Charlwood et al. [6], Blatter et al. [12] and Khoja et al. [18]. From a nursing management perspective, reliance on average staffing levels or a single RN staffing ratio may therefore obscure clinically meaningful variation in when staff are available, how the nursing workforce is composed and how staffing is organised in relation to changing resident care needs.

Endogeneity and residual confounding remain important limitations of the available evidence. Staffing levels are not randomly distributed across nursing homes and may vary according to resident case mix, facility resources, organisational conditions and previous quality performance. These same factors can also influence resident outcomes, making it difficult to isolate the contribution of staffing alone. Konetzka et al. [14] addressed staffing endogeneity using facility fixed‐effects and instrumental‐variable methods, providing stronger grounds for causal interpretation than most observational studies in this field. However, the methodological strength of one study should not be extended to a wider literature that remains largely observational. Pradhan et al. [33] also showed that neighbourhood social deprivation was related to staffing patterns and pathways to quality, suggesting that the broader social and organisational context may shape observed staffing–outcome relationships. The findings should therefore be interpreted cautiously. In most studies, the evidence supports an association between staffing and outcomes rather than a causal effect.

Workforce sustainability is an important consideration in this review, although its relationship with resident outcomes should be interpreted with care. White et al. [4] found that more favourable nursing‐home work environments were associated with fewer pressure ulcers and hospitalisations, as well as lower levels of RN burnout and job dissatisfaction. Cho et al. [5] reported a similar pattern, showing that better work environments were associated with higher resident quality of life while also identifying substantial levels of RN job dissatisfaction, burnout and intention to leave. These studies indicate that the work environment and workforce stability may affect how effectively nursing homes sustain consistent and responsive care. However, they do not demonstrate a clear causal sequence in which workload leads to burnout or turnover and, in turn, to poorer resident outcomes. In this review, workforce sustainability is therefore considered an important organisational context for understanding RN workload and resident outcomes, rather than a confirmed causal mechanism.

The current evidence does not justify presenting staffing or work‐environment strategies as established management interventions. Most primary studies were observational and focused on associations rather than on the effects of deliberately implemented organisational changes. For nursing‐home management, a more appropriate interpretation is to consider RN HPRD alongside skill mix, agency use, supervisory coverage and characteristics of the work environment [4–6, 12, 18]. Broader considerations identified across the synthesis include resident acuity, staffing continuity and workload intensity. These factors may be useful for workload monitoring and local evaluation, but the available evidence is not strong enough to support them as established or universally applicable management prescriptions.

Seven studies were retained as contextual evidence because they contributed relevant organisational, workforce, work‐organisation and professional‐role perspectives but did not provide sufficiently separable RN‐specific resident‐outcome data. Anderson et al. [24] contributed evidence on management practice and organisational context, while Bowers et al. [3] examined licensed‐nurse time pressure and work organisation. Chen et al. [25] assessed staff shortages, overall staffing levels and resident outcomes using mixed staffing measures, and Sinha et al. [26] examined staffing instability, turnover and quality. Munkeby et al. [10] focused on RN stress of conscience and workforce wellbeing, Tchouaket et al. [27] evaluated a nurse‐practitioner model and associated costs, and Väisänen et al. [11] examined stress and time pressure among mixed nursing personnel. These studies were used to provide context for interpreting management practices, workforce conditions, staffing instability, work organisation and professional roles. However, because they did not isolate a clear RN‐specific relationship with resident outcomes, they were not included as primary evidence in the main synthesis.

Several evidence gaps remained. Future research should distinguish structural staffing capacity from experienced RN workload and organisational context, as staffing numbers alone do not capture interruptions, documentation burden, cognitive and emotional demands, supervisory responsibilities, workload peaks or fluctuations in care demand. Longitudinal and shift‐level studies are needed to examine how these pressures change over time and relate to resident outcomes. Greater attention should also be given to resident‐reported outcomes, including quality of life and care experience. Studies with clearer temporal ordering and stronger approaches to nonlinearity, confounding and endogeneity would provide a more robust basis for staffing and management decisions [6, 14, 15]. For nursing management, staffing benchmarks should be interpreted in relation to local context rather than as fixed prescriptions. Managers should consider staffing configuration, skill mix, agency use, supervisory coverage, work environment, resident acuity, staffing continuity and workload intensity alongside numerical staffing measures [4–6, 12, 18]. This broader interpretation is more consistent with the observational and heterogeneous nature of the available evidence.

## 6. Limitations

Several limitations should be considered when interpreting the findings of this review. First, the source‐level audit identified limitations in the sensitivity of the original search, as some eligible RN‐specific studies had not been retrieved. We therefore broadened the search strategy and reran all five prespecified databases on 4 September 2026. Despite this update, we cannot exclude the possibility that additional eligible studies were not identified. Second, we used a two‐layer evidence structure. Twenty studies underwent full RN‐specific extraction and formed the primary synthesis, whereas seven studies underwent restricted contextual extraction because they did not provide a sufficiently separable RN‐specific relationship with resident outcomes. Although this approach allowed us to retain relevant organisational and workforce evidence, it required a clear distinction between primary evidence and contextual interpretation. Third, most primary studies were observational; several relied on facility‐level administrative data and outcome definitions varied considerably across studies. These features limited causal interpretation and comparability across the evidence base. Fourth, consistent with the purpose of a scoping review, we did not conduct a meta‐analysis because our objective was to map and interpret the available evidence rather than estimate a pooled effect. Finally, expert consultation helped refine the interpretation, terminology and practical relevance of the synthesis, but it did not constitute independent validation of the included evidence.

## 7. Conclusion

This scoping review mapped the available evidence on RN workload, staffing measures, resident outcomes, workforce sustainability and the wider nursing‐management context in nursing homes. The findings show that RN workload cannot be understood through staffing ratios or RN HPRD alone. It is shaped by how staffing is organised, the composition of the care team, the demands placed on RNs and the conditions in which care is delivered. Across the included studies, greater RN availability, a more appropriate skill mix and more favourable work environments were often associated with better resident outcomes. However, these relationships were not consistent across all outcomes or settings and varied according to timing, staffing configuration and organisational context.

The evidence did not support a universal RN staffing threshold or indicate that increasing RN hours alone would consistently improve resident outcomes. Staffing measures should therefore be interpreted alongside resident acuity, staffing continuity, team composition, supervisory responsibilities, workload intensity and the work environment. The main contribution of this review was to distinguish numerical staffing from the broader construct of RN workload, providing a clearer basis for workforce monitoring, nursing management and future research on the conditions under which RN staffing is associated with better resident outcomes.

## 8. Implications for Nursing Management and Future Directions

HPRD and nurse‐to‐resident ratios can be used as indicators of staffing capacity, although they do not provide a complete measure of RN workload. A broader assessment may also consider resident acuity, staffing continuity, agency use, supervisory responsibilities, documentation demands, workload intensity and the work environment [4–6, 12, 18]. Monitoring these factors alongside resident and workforce outcomes may provide additional information when evaluating local staffing arrangements and emerging workload pressures.

Workforce sustainability may also warrant consideration within nursing management and quality improvement. Supportive leadership, clear roles, stable staffing and manageable supervisory responsibilities may influence how effectively RN resources are used and sustained over time [4, 5]. However, the available evidence does not establish a single management strategy or staffing threshold that can be applied across nursing homes. Staffing policies and organisational changes may therefore be evaluated in relation to local context, including resident acuity, skill mix, staffing configuration and organisational resources.

Future research could extend beyond comparisons of staffing ratios alone. Longitudinal and shift‐level studies, linked nurse‐resident datasets, validated multidimensional workload measures, quasi‐experimental designs and natural experiments following staffing‐policy changes may strengthen understanding of temporal, nonlinear and potentially causal relationships [6, 14, 15]. Greater attention may also be given to resident‐reported outcomes, particularly quality of life and care experience and to evidence from underrepresented health systems. Such research could clarify when and under what organisational conditions RN staffing and workload are associated with resident and workforce outcomes.

## Funding

This Open Access publication was supported by Nazarbayev University under the transformative agreement with Wiley.

## Ethics Statement

This scoping review synthesised published literature and did not involve recruitment of human participants, collection of identifiable personal data, or access to nonpublic clinical records; ethics approval was therefore not required for the literature‐review component. The four‐expert consultation was limited to methodological and interpretive feedback on the synthesis, terminology, conceptual framework and management relevance and was not analysed as human‐participant research data.

## Conflicts of Interest

The authors declare no conflicts of interest.

## Supporting Information

Additional supporting information can be found online in the Supporting Information section.

## Supporting information


**Supporting Information 1** Supporting Table 1: Completed PRISMA‐ScR checklist reporting the location of relevant scoping‐review items, including the updated search, two‐layer data‐charting process, 20‐study RN‐specific primary synthesis, separately reported seven‐study contextual evidence layer, and updated Figure 1.

**Supporting Information 2** Supporting Table 2: Source‐level RN‐specific eligibility re‐audit and contextual data‐charting record. Part A reports the 32 previously extended‐retrieval/full‐text records. Four met the final RN‐specific eligibility criterion: Alexander [28], Cho et al. [29], Konetzka et al. [14] and Mukamel et al. [15]; 28 did not. Because Konetzka et al. [14] was already included in the original evidence set, only Alexander [28], Cho et al. [29] and Mukamel et al. [15] were newly recovered unique studies. Part B documents the seven studies from the original review that did not meet the RN‐specific primary criterion and records the contextual information retained from each. These studies were not included in the RN‐specific primary synthesis and were not used to support RN‐specific associations with resident outcomes.

**Supporting Information 3** Supporting Table 3: Database‐specific search strategies for PubMed, ScienceDirect, Scopus, Web of Science Core Collection and CINAHL (EBSCOhost), together with the final screening and data‐extraction rules. The updated search was conducted on 4 September 2026 using nursing‐home or residential long‐term‐care setting, RN and workload or staffing as the three core search concepts, with resident outcomes assessed during eligibility screening. Full data extraction was restricted to the 20 RN‐specific primary studies. The seven contextual studies underwent restricted contextual data charting and were not counted in the primary synthesis.

## Data Availability

The data that support the findings of this study are available from the corresponding author upon reasonable request.

## References

[1] World Health Organization , Global Strategy and Action Plan on Ageing and Health, 2017, World Health Organization.

[2] Park Y. and Min D. , Registered Nurses’ Perspective of Systemic Factors Affecting Nursing Home Care Quality Decline: a Qualitative Descriptive Study, Nursing Open. (2023) 10, no. 3, 1900–1908, 10.1002/nop2.1474. PMC9912408 36373815

[3] Bowers B. J. , Lauring C. , and Jacobson N. , How Nurses Manage Time and Work in long-term Care, Journal of Advanced Nursing. (2001) 33, no. 4, 484–491, 10.1046/j.1365-2648.2001.01686.x. 11251736

[4] White E. M. , Aiken L. H. , Sloane D. M. , and McHugh M. D. , Nursing Home Work Environment, Care Quality, Registered Nurse Burnout and Job Dissatisfaction, Geriatric Nursing. (2020) 41, no. 2, 158–164, 10.1016/j.gerinurse.2019.08.007. PMC7051884 31488333

[5] Cho E. , Min D. , Heo S.-J. , Lee K. , and Kim H. , Effects of Registered Nurses’ Staffing Levels, Work Environment and Education Levels on Nursing Home Residents’ Quality of Life and Nurse Outcomes, Journal of Clinical Nursing. (2023) 32, no. 17-18, 6494–6503, 10.1111/jocn.16689. 36932041

[6] Charlwood A. , Valizade D. , Winton Schreuders L. et al., Workforce Thresholds and the Non-linear Association Between Registered Nurse Staffing and Care Quality in long-term Residential Care: a Retrospective Longitudinal Study of English Care Homes with Nursing, International Journal of Nursing Studies. (2024) 157, 10.1016/j.ijnurstu.2024.104815. 38905748

[7] Shin J. H. , Renaut R. A. , Reiser M. , Lee J. Y. , and Tang T. Y. , Increasing Registered Nurse Hours per Resident Day for Improved Nursing Home Residents’ Outcomes Using a Longitudinal Study, International Journal of Environmental Research and Public Health. (2021) 18, no. 2, 10.3390/ijerph18020402. PMC7825529 33419183

[8] Choi S. , Cho E. , Kim E. , Lee K. , and Chang S. J. , Effects of Registered Nurse Staffing Levels, Work Environment, and Education Levels on Adverse Events in Nursing Homes, Scientific Reports. (2021) 11, no. 1, 10.1038/s41598-021-00946-8. PMC8563772 34728676

[9] Jutkowitz E. , Landsteiner A. , Ratner E. et al., Effects of Nurse Staffing on Resident Outcomes in Nursing Homes: a Systematic Review, Journal of the American Medical Directors Association. (2023) 24, no. 1, 75–81.e11, 10.1016/j.jamda.2022.11.002. 36470321

[10] Munkeby H. , Bratberg G. , and Devik S. A. , Registered Nurses’ Exposure to High Stress of Conscience in long-term Care, Nursing Ethics. (2023) 30, no. 7-8, 1011–1024, 10.1177/09697330231167542. PMC10710004 37163482

[11] Väisänen V. , Ruotsalainen S. , Hietapakka L. , Sulander J. , and Sinervo T. , The Role of Workday Characteristics on Perceived Stress and Time Pressure Among Nurses in Finnish long-term Care – a cross-sectional Study, BMC Health Services Research. (2024) 24, no. 1, 10.1186/s12913-024-11294-4. PMC11295524 39095796

[12] Blatter C. , Simon M. , and Zúñiga F. , Staffing Patterns and Pressure Injury Incidence in Swiss long-term Care: a time-to-event Study, Journal of the American Medical Directors Association. (2026) 27, no. 2, 10.1016/j.jamda.2025.106025. 41365362

[13] Blatter C. , Osińska M. , Simon M. , and Zúñiga F. , The Relationship Between Nursing Home Staffing and Resident Safety Outcomes: a Systematic Review of Reviews, International Journal of Nursing Studies. (2024) 150, 10.1016/j.ijnurstu.2023.104641. 37992653

[14] Konetzka R. T. , Stearns S. C. , and Park J. , The staffing–outcomes Relationship in Nursing Homes, Health Services Research. (2008) 43, no. 3, 1025–1042, 10.1111/j.1475-6773.2007.00803.x. PMC2442239 18454779

[15] Mukamel D. B. , Saliba D. , Ladd H. , and Konetzka R. T. , Associations Between Daily Nurse Staffing Levels and Daily Hospitalizations and ED Visits in Nursing Homes, Journal of the American Medical Directors Association. (2022) 23, no. 11, 1793–1799.e3, 10.1016/j.jamda.2022.06.030. PMC9955393 35948066

[16] Peters M. D. J. , Marnie C. , Tricco A. C. et al., Updated Methodological Guidance for the Conduct of Scoping Reviews, JBI Evidence Synthesis. (2020) 18, no. 10, 2119–2126, 10.11124/JBIES-20-00167. 33038124

[17] Yoon J. M. , Trinkoff A. M. , Galik E. et al., Nurse Staffing and Deficiency of Care for Inappropriate Psychotropic Medication Use in Nursing Home Residents with Dementia, Journal of Nursing Scholarship. (2022) 54, no. 6, 728–737, 10.1111/jnu.12776. 35388951

[18] Khoja J. S. , MacLean K. G. , Lee Y. , Joshi R. , Daiello L. A. , and Berry S. D. , Falls in the Nursing Home: the Impact of Staffing Levels and Agency Staff Use on Injurious Falls, Journal of the American Medical Directors Association. (2026) 27, no. 3, 10.1016/j.jamda.2025.106083. PMC12925139 41545006

[19] Yang B. K. , Carter M. W. , Trinkoff A. M. , and Nelson H. W. , Nurse Staffing and Skill Mix Patterns in Relation to Resident Care Outcomes in US Nursing Homes, Journal of the American Medical Directors Association. (2021) 22, no. 5, 1081–1087.e1, 10.1016/j.jamda.2020.09.009. 33132015

[20] Abdulai A. S. B. , Nursing Home Staffing Levels and Resident Health Outcomes: Is the Role of the Physical Therapist Undervalued?, Journal of the American Medical Directors Association. (2025) 26, no. 2, 10.1016/j.jamda.2024.105422. 39706578

[21] Arksey H. and O’Malley L. , Scoping Studies: towards a Methodological Framework, International Journal of Social Research Methodology. (2005) 8, no. 1, 19–32, 10.1080/1364557032000119616.

[22] Tricco A. C. , Lillie E. , Zarin W. et al., PRISMA Extension for Scoping Reviews (PRISMA-ScR): Checklist and Explanation, Annals of Internal Medicine. (2018) 169, no. 7, 467–473, 10.7326/M18-0850. 30178033

[23] Page M. J. , McKenzie J. E. , Bossuyt P. M. et al., The PRISMA 2020 Statement: an Updated Guideline for Reporting Systematic Reviews, BMJ. (2021) 372, 10.1136/bmj.n71. PMC8005924 33782057

[24] Anderson R. A. , Issel L. M. , and McDaniel R. R.Jr., Nursing Homes as Complex Adaptive Systems: Relationship Between Management Practice and Resident Outcomes, Nursing Research. (2003) 52, no. 1, 12–21, 10.1097/00006199-200301000-00003. PMC1993902 12552171

[25] Chen M. , Goodwin J. S. , Bailey J. E. , Bowblis J. R. , Li S. , and Xu H. , Longitudinal Associations of Staff Shortages and Staff Levels with Health Outcomes in Nursing Homes, Journal of the American Medical Directors Association. (2023) 24, no. 11, 1755–1760.e7, 10.1016/j.jamda.2023.04.017. PMC10826288 37263319

[26] Sinha S. , Mukamel D. B. , Saliba D. , Ladd H. , and Konetzka R. T. , New Dimensions of Staffing Patterns in Nursing Homes and Nursing Home Quality: Comparing Staffing Instability to Staffing Turnover, Journal of the American Medical Directors Association. (2023) 24, no. 8, 1099–1105.e7, 10.1016/j.jamda.2023.04.009. PMC10524116 37247823

[27] Tchouaket E. , Kilpatrick K. , and Jabbour M. , Effectiveness for Introducing Nurse Practitioners in Six long-term Care Facilities in Québec, Canada: a cost-savings Analysis, Nursing Outlook. (2020) 68, no. 5, 611–625, 10.1016/j.outlook.2020.06.002. 32713732

[28] Alexander G. L. , An Analysis of Nursing Home Quality Measures and Staffing, Quality Management in Health Care. (2008) 17, no. 3, 242–251, 10.1097/01.qmh.0000326729.78331.c5. PMC3006165 18641507

[29] Cho E. , Kim I. S. , Lee T. W. , Kim G. S. , Lee H. , and Min D. , Effects of Registered Nurse Staffing on Quality of Care and Resident Outcomes in Nursing Homes, Geriatric Nursing. (2020) 41, no. 6, 685–691, 10.1016/j.gerinurse.2020.04.001. 32386999

[30] Chappell V. , Kirkham J. , and Seitz D. P. , Association Between long-term Care Facility Staffing Levels and Antipsychotic Use in US long-term Care Facilities, Journal of the American Medical Directors Association. (2022) 23, no. 11, 1787–1792.e1, 10.1016/j.jamda.2022.06.029. 35926573

[31] Harrington C. , Ross L. , Chapman S. , Halifax E. , Spurlock B. , and Bakerjian D. , Nurse Staffing and Coronavirus Infections in California Nursing Homes, Policy, Politics, & Nursing Practice. (2020) 21, no. 3, 174–186, 10.1177/1527154420938707. 32635838

[32] Lee J. , Shin J. H. , Lee K. H. , Harrington C. A. , and Jung S. O. , Staffing Levels and COVID-19 Infections and Deaths in Korean Nursing Homes, Policy, Politics, & Nursing Practice. (2022) 23, no. 1, 15–25, 10.1177/15271544211056051. PMC8801339 34939511

[33] Pradhan R. , Lord J. , Orewa G. , Davlyatov G. , and Weech-Maldonado R. , How Neighborhood Social Deprivation is Associated with Nursing Home Quality: a Structure, Process, Outcome Model, Innovation in Aging. (2025) 9, no. 2, 10.1093/geroni/igaf004. PMC11831227 39963524

[34] Shin J. H. , Jung S. O. , and Min E. J. , Factors Influencing Hospitalization of Nursing Home Residents in Korea Using Regularized Negative Binomial Regression, Policy, Politics, & Nursing Practice. (2024) 25, no. 3, 141–151, 10.1177/15271544241259427. 38874520

