# Supplementary material for: Registered Nurse Workload and Resident Outcomes in Nursing Homes: A Scoping Review of Workforce Sustainability and Nursing Management
Source: J Nurs Manag. 2026 Oct 13;2026:6082303. doi: 10.1155/jonm/6082303 (PMC13662227; doi:10.1155/jonm/6082303)
Supplement: Supplementary file 1 [file JONM-2026-6082303-s001.docx]

**Supplementary Table 3. Revised database-specific electronic search strategies for the final update rerun**

**Final update-search status.** The five prespecified databases: PubMed, ScienceDirect, Scopus, Web of Science Core Collection, and CINAHL (EBSCOhost) were rerun on 4 September 2026. The revised search focused on nursing-home or residential long-term-care settings, registered nurses, and workload or staffing, with resident outcomes assessed during eligibility screening. No duplicate records were identified (n = 0). Following screening and source reconciliation, 20 unique studies were included in the RN-specific synthesis. Seven studies did not meet the primary eligibility criteria and were retained only as contextual evidence.

| **Database** | **Revised search strategy** | **Limits** |
| --- | --- | --- |
| PubMed | (("Nursing Homes"[Mesh] OR "Long-Term Care"[Mesh] OR "nursing home*"[tiab] OR "long-term care facilit*"[tiab] OR "care home*"[tiab] OR "residential aged care"[tiab]) AND ("Nurses"[Mesh] OR "registered nurse*"[tiab] OR RN[tiab] OR RNs[tiab] OR "professional nurse*"[tiab]) AND ("Workload"[Mesh] OR workload[tiab] OR staffing[tiab] OR "staffing level*"[tiab] OR "hours per resident day"[tiab] OR HPRD[tiab] OR "skill mix"[tiab] OR "work environment"[tiab] OR "staffing stability"[tiab] OR "agency staff*"[tiab] OR turnover[tiab] OR continuity[tiab])) | Publication date: 1 Jan 2000 to 4 Sep 2026; English. Article-type eligibility was applied during screening. |
| Scopus | TITLE-ABS-KEY(("nursing home*" OR "long-term care facilit*" OR "care home*" OR "residential aged care") AND ("registered nurse*" OR RN OR RNs OR "professional nurse*") AND (workload OR staffing OR "staffing level*" OR "hours per resident day" OR HPRD OR "skill mix" OR "work environment" OR "staffing stability" OR "agency staff*" OR turnover OR continuity)) | Publication year: 2000–2026; final search date: 4 Sep 2026; English. |
| Web of Science Core Collection | TS=(("nursing home*" OR "long-term care facilit*" OR "care home*" OR "residential aged care") AND ("registered nurse*" OR RN OR RNs OR "professional nurse*") AND (workload OR staffing OR "staffing level*" OR "hours per resident day" OR HPRD OR "skill mix" OR "work environment" OR "staffing stability" OR "agency staff*" OR turnover OR continuity)) | Timespan: 2000 to 4 Sep 2026; English. |
| CINAHL (EBSCOhost) | S1: (MH "Nursing Homes+") OR (MH "Long Term Care+") OR TI ("nursing home*" OR "long-term care facilit*" OR "care home*" OR "residential aged care") OR AB ("nursing home*" OR "long-term care facilit*" OR "care home*" OR "residential aged care") S2: (MH "Nurses+") OR TI ("registered nurse*" OR RN OR RNs OR "professional nurse*") OR AB ("registered nurse*" OR RN OR RNs OR "professional nurse*") S3: (MH "Workload+") OR (MH "Personnel Staffing and Scheduling+") OR TI (workload OR staffing OR "staffing level*" OR "hours per resident day" OR HPRD OR "skill mix" OR "work environment" OR "staffing stability" OR "agency staff*" OR turnover OR continuity) OR AB (workload OR staffing OR "staffing level*" OR "hours per resident day" OR HPRD OR "skill mix" OR "work environment" OR "staffing stability" OR "agency staff*" OR turnover OR continuity) S4: S1 AND S2 AND S3 | Published date: 1 Jan 2000 to 4 Sep 2026; English. |
| ScienceDirect | ("nursing home" OR "nursing homes" OR "long-term care facility" OR "long-term care facilities" OR "care home" OR "care homes" OR "residential aged care") AND ("registered nurse" OR "registered nurses" OR RN OR RNs OR "professional nurse") AND (workload OR staffing OR "staffing level" OR "staffing levels" OR "hours per resident day" OR HPRD OR "skill mix" OR "work environment" OR "staffing stability" OR "agency staff" OR turnover OR continuity) | Date range: 2000 to 4 Sep 2026; English; Research articles. |

**Eligibility and extraction criteria.** The RN-specific synthesis included studies conducted in nursing-home or residential long-term-care settings that reported a clearly separable RN workload or staffing measure and at least one eligible resident outcome. Full data extraction was completed for the 20 studies that met these criteria, covering study characteristics, RN-specific exposure, resident outcomes, analytical approach, and key findings. Seven studies did not meet the primary eligibility criteria but were retained as contextual evidence. For these studies, extraction was limited to study context, reason for exclusion from the primary synthesis, relevant contextual domain, and findings that informed interpretation. Their effect estimates were not included in the RN-specific resident-outcome synthesis.
