# Supplementary material for: Registered Nurse Workload and Resident Outcomes in Nursing Homes: A Scoping Review of Workforce Sustainability and Nursing Management
Source: J Nurs Manag. 2026 Oct 13;2026:6082303. doi: 10.1155/jonm/6082303 (PMC13662227; doi:10.1155/jonm/6082303)
Supplement: Supplementary file 3 [file JONM-2026-6082303-s003.docx]

**Supplementary Table 2. Source-level RN-specific eligibility re-audit and contextual data-charting record**

**Note.** Part A re-audits the 32 records previously subjected to extended retrieval/full-text assessment against the revised RN-specific primary-synthesis criterion: English-language peer-reviewed primary research published from 1 January 2000 through 4 September 2026; nursing-home/residential long-term-care context; a separable registered nurse (RN)-specific workload or staffing exposure/finding; and at least one resident health, safety, service-use, medication-related quality, psychosocial, or quality-of-life outcome. Four of the 32 records met this criterion: Alexander (2008), Cho et al. (2020), Konetzka et al. (2008), and Mukamel et al. (2022), and 28 did not. Because Konetzka et al. (2008) was already included in the original evidence set, only three were newly added unique studies. Part B documents the seven original studies excluded from the RN-specific primary synthesis and records the restricted contextual data extracted from each. Part A is an audit trail for these 32 records only and does not represent the complete 20-study primary evidence set. As reported in the manuscript, the final RN-specific primary synthesis comprises 9 retained studies from the original 16-study evidence set, 8 additional RN-specific studies identified through the sensitivity re-audit, and 3 newly recovered unique studies from the 32-record re-examination, for 20 unique primary studies in total. No duplicate records were identified in the 4 September 2026 update-search set (n = 0). RN = registered nurse; CNA = certified nursing assistant; LPN = licensed practical nurse; NH = nursing home; LTC = long-term care; HPRD = hours per resident day.

**Part A. Re-audit of 32 extended-retrieval/full-text records**

| **No.** | **Full citation** | **Primary eligibility assessment** | **Secondary assessment / rationale** | **Decision** |
| --- | --- | --- | --- | --- |
| 1 | Ahn, J., Yang, Y., & Park, G. (2024). Advancing elderly diabetes care: Exploring the usability and acceptance of continuous glucose monitoring (CGM). Geriatric Nursing, 59, 15–25. https://doi.org/10.1016/j.gerinurse.2024.06.041 | No RN-specific workload or staffing exposure is examined; the study evaluates CGM usability and acceptance among older adults with diabetes. | The study was conducted in a community-based public health centre rather than a nursing home or residential long-term-care setting, and it reports no eligible RN-specific exposure linked to a resident outcome. | Exclude |
| 2 | Alexander, G. L., Madsen, R., & Newton, M. (2017). Analyzing Change in Nursing Home Information Technology Sophistication: A 2-Year Survey. Journal of Gerontological Nursing, 43(1), 17–21. https://doi.org/10.3928/00989134-20161215-05 | The study examines nursing-home information-technology sophistication and adoption, not an RN-specific workload or staffing exposure linked to a resident outcome. | Respondents are nursing-home administrators, and no separable RN-specific workload or staffing finding is reported. | Exclude |
| **3** | Alexander, G. L. (2008). An Analysis of Nursing Home Quality Measures and Staffing. Quality Management in Health Care, 17(3), 242–251. https://doi.org/10.1097/01.qmh.0000326729.78331.c5 | RN HPRD is analysed as a distinct exposure alongside LPN and CNA HPRD; RN-specific estimates are therefore separable under the revised eligibility criterion. | The setting is nursing homes and the outcomes are resident quality measures (continence, ADL decline, pain and pressure ulcers). Facility-level HPRD is an eligible staffing-related workload indicator in the revised review framework. | Include |
| **4** | Anderson, R. A., Hsieh, P.-C., & Su, H.-F. (1998). Resource allocation and resident outcomes in nursing homes: Comparisons between the best and worst. Research in Nursing & Health, 21(4), 297–313. https://doi.org/10.1002/(sici)1098-240x(199808)21:4<297::aid-nur3>3.0.co;2-a | Outside the publication window: 1998; eligibility begins 1 January 2000. | RN staffing is analysed separately, but the 1998 publication falls outside the prespecified 2000–2026 window. | Exclude |
| 5 | Bäckström, J., Fogelberg, J., Gabrielsson, G., Lindberg, M., Arving, C., & Hellström, K. (2020). Reliability and internal consistency of the Swedish version of the MAastrIcht Nurses Activities INventory (MAINtAIN(S)) - A pilot testing of the tool. Geriatric nursing (New York, N.Y.), 41(6), 790–803. https://doi.org/10.1016/j.gerinurse.2020.04.012 | The study includes nursing assistants only and therefore provides no separable RN-specific workload or staffing exposure. | It evaluates the reliability and internal consistency of a measurement instrument and does not report an eligible resident outcome linked to an RN-specific exposure. | Exclude |
| 6 | Barbosa, M. M., Teixeira, L., Edvardsson, D., Paul, C., Yanguas, J., & Afonso, R. M. (2023). Adaptation and validation of the Person-centered Care Assessment Tool (P-CAT) to the Portuguese population. International journal of older people nursing, 18(2), e12522. https://doi.org/10.1111/opn.12522 | The study uses a mixed-staff sample, and RN-specific findings are not reported separately. | It validates the Person-centred Care Assessment Tool and does not examine an RN-specific workload or staffing exposure linked to an eligible resident outcome. | Exclude |
| 7 | Bayram, S., & Akkaş, Ö. A. (2024). Adaptation of the Australian National University Alzheimer's Disease Risk Index-Short Form (ANU-ADRI-SF) into Turkish. International Journal of Older People Nursing, 19, e12608. https://doi.org/10.1111/opn.12608 | Study conducted with community-based adults recruited from hospital waiting rooms and social facilities; no nursing home or long-term care setting involved at any stage | Participants are community-dwelling adults aged 40 and over being screened for Alzheimer's disease risk; no registered nurses or nursing home residents involved. Study adapts an Alzheimer's disease risk index into Turkish; no measurement of nurses' workload or resident care outcomes | Exclude |
| 8 | Bell, C. M., & Redelmeier, D. A. (2001). Mortality among Patients Admitted to Hospitals on Weekends as Compared with Weekdays. New England Journal of Medicine, 345(9), 663–668. https://doi.org/10.1056/nejmsa003376 | The study was conducted exclusively in acute-care hospitals, outside the nursing-home/residential long-term-care context required for inclusion. | No RN-specific nursing-home workload or staffing exposure is analysed; reduced weekend staffing is mentioned only as a possible explanation in the Discussion rather than measured as the study exposure. | Exclude |
| 9 | Bennesved, A., Bremer, A., Svensson, A., Rantala, A., & Holmberg, M. (**2024**). Ambulance clinicians’ understanding of older patients’ self-determination: A vignette study. Nursing Ethics, 31(2–3), 342–354. https://doi.org/10.1177/09697330231196230 | Prehospital ambulance setting, not a nursing-home or residential LTC setting. | No RN-specific nursing-home workload or staffing exposure is linked to an eligible resident outcome. | Exclude |
| 10 | Bowblis, J. R., & Hyer, K. (2013). Nursing Home Staffing Requirements and Input Substitution: Effects on Housekeeping, Food Service, and Activities Staff. Health Services Research, 48(4), 1539–1550. https://doi.org/10.1111/1475-6773.12046 | The study examines state-level nursing-home staffing requirements and substitution across staffing inputs; its primary outcome is support-staff employment rather than an eligible resident outcome. | RN, LPN and CNA staffing are treated within the regulatory staffing framework rather than as a separable RN-specific exposure linked to a resident outcome. | Exclude |
| 11 | Bowblis, J. R., & Roberts, A. R. (2020). Cost-Effective Adjustments to Nursing Home Staffing to Improve Quality. Medical Care Research and Review, 77(3), 274–284. https://doi.org/10.1177/1077558718778081 | Facility staffing inputs are related to regulatory deficiency scores; no separable RN-specific exposure is linked to an eligible resident outcome. | HPRD is eligible, but RN effects cannot be separated from other nursing categories; deficiency scores are regulatory metrics rather than direct resident outcomes. | Exclude |
| 12 | Brewster, G. S., Hirschman, K. B., Riegel, B. J., Hanlon, A. L., Huang, L., McPhillips, M. V., Abbott, K. M., & Naylor, M. D. (2019). Association of health related quality of life domains with daytime sleepiness among elderly recipients of long-term services and supports. Geriatric Nursing, 40(4), 417–423. https://doi.org/10.1016/j.gerinurse.2019.01.006 | The study examines health-related quality of life and daytime sleepiness among recipients of long-term services and supports; no RN-specific workload or staffing exposure is analysed. | The sample spans nursing homes, assisted-living communities, and home/community-based services, with no separable nursing-home RN exposure-outcome analysis. | Exclude |
| **13** | Cho, E., Kim, I. S., Lee, T. W., Kim, G. S., Lee, H., & Min, D. (2020). Effects of registered nurse staffing on quality of care and resident outcomes in nursing homes. Geriatric Nursing, 41(6), 685–691. https://doi.org/10.1016/j.gerinurse.2020.04.001 | The study directly evaluates registered nurse staffing in South Korean nursing homes and reports RN-specific staffing as the exposure. | Resident outcomes include mortality and nursing-home quality ratings; the study therefore meets the revised RN-specific staffing and resident-outcome criterion. | Include |
| 14 | Dahlkvist, E., Engström, M., & Nilsson, A. (2020). Residents’ use and perceptions of residential care facility gardens: A behaviour mapping and conversation study. International Journal of Older People Nursing, 15(1), e12283. https://doi.org/10.1111/opn.12283 | Resident garden-use study; no RN-specific workload or staffing exposure. | Staff appear only as facilitators of outdoor access; no separable RN exposure is linked to an eligible resident outcome. | Exclude |
| 15 | Dietermann, K., Winter, V., Schneider, U., & Schreyögg, J. (2021). The impact of nurse staffing levels on nursing-sensitive patient outcomes: a multilevel regression approach. The European Journal of Health Economics, 22(5), 833–846. https://doi.org/10.1007/s10198-021-01292-2 | The study is conducted exclusively in German acute care hospitals, a setting directly listed under the review's exclusion criteria. No nursing home or long-term care setting is examined. | The study is based on acute-care hospital units and therefore fails the review context criterion. Its staffing measure also combines RNs and assistant nurses rather than providing the nursing-home RN-specific evidence required for this review. | Exclude |
| 16 | Erlang, A. S., Schjødt, K., Linde, J. K. S., & Jensen, A. L. (2021). An observational study of older patients' experiences of involvement in discharge planning. Geriatric Nursing, 42(4), 855–862. https://doi.org/10.1016/j.gerinurse.2021.04.002 | Study conducted exclusively in an acute care hospital (Department of Endocrinology and Internal Medicine, Aarhus University Hospital), a setting directly listed under the review's exclusion criteria. | The study examines older medical inpatients and discharge planning in an acute-care hospital. No RN-specific workload or staffing exposure is analysed. | Exclude |
| 17 | Gifford, A., Green, R. K., Jacobsohn, G. C., Cochran, A. L., Caprio, T. V., Cushman, J. T., Jones, C. M. C., Kind, A. J. H., Lohmeier, M., & Shah, M. N. (2022). Scalar Assessment of the Family Caregiver Activation in Transitions Tool: An Exploratory Factor Analysis. Journal of Gerontological Nursing, 48(12), 35–42. https://doi.org/10.3928/00989134-20221107-04 | The study evaluates family-caregiver activation and does not analyse an RN-specific workload or staffing exposure. | Data were collected in emergency departments at two academic medical centres, not in nursing homes or residential long-term-care settings, and no eligible RN-specific exposure-outcome relationship is reported. | Exclude |
| 18 | Halifax, E., & Harrington, C. (2024). Proposed minimum nurse staffing levels in nursing homes. Policy, Politics, & Nursing Practice, 25(2), 67–69. https://doi.org/10.1177/15271544241237653 | The article is a Guest Editorial/opinion piece rather than eligible peer-reviewed primary research and is excluded by study type. | Study type is independently disqualifying; the article does not provide an eligible primary-study dataset for the RN-specific synthesis. | Exclude |
| 19 | Harrington, C., Kovner, C., Mezey, M., Kayser-Jones, J., Burger, S., Mohler, M., Burke, R., & Zimmerman, D. (2000). Experts recommend minimum nurse staffing standards for nursing facilities in the United States. The Gerontologist, 40(1), 5–16. https://doi.org/10.1093/geront/40.1.5 | The article is an expert consensus/policy paper rather than eligible primary empirical research and is therefore excluded by study type. | Not applicable; exclusion rests on study type. | Exclude |
| 20 | Hyer, K., Thomas, K. S., Branch, L. G., Harman, J. S., Johnson, C. E., & Weech-Maldonado, R. (2011). The influence of nurse staffing levels on quality of care in nursing homes. The Gerontologist, 51(5), 610–616. https://doi.org/10.1093/geront/gnr050 | RN staffing is not separable because RNs are combined with LPNs in a single licensed-nurse variable; no RN-specific staffing estimate can therefore be extracted. | CNA staffing is analysed separately, but CNA findings do not satisfy the RN-specific population/exposure criterion. | Exclude |
| 21 | Irvine, B., Billow, M. B., Gates, D. M., Fitzwater, E. L., Seeley, J. R., & Bourgeois, M. (2012). An Internet Training to Reduce Assaults in Long-Term Care. Geriatric Nursing, 33(1), 28–40. https://doi.org/10.1016/j.gerinurse.2011.10.004 | The study evaluates an Internet-based aggression-management intervention for nurse aides/CNAs and provides no RN-specific workload or staffing exposure. | The intervention and outcomes concern nurse aides rather than a separable RN-specific exposure linked to an eligible resident outcome. | Exclude |
| 22 | Jiwani, R., Dennis, B., Bess, C., Monk, S., Meyer, K., Wang, J., & Espinoza, S. (2021). Assessing acceptability and patient experience of a behavioral lifestyle intervention using fitbit technology in older adults to manage type 2 diabetes amid COVID-19 pandemic: A focus group study. Geriatric Nursing, 42(1), 57–64. https://doi.org/10.1016/j.gerinurse.2020.11.007 | The study evaluates a Fitbit-based diabetes self-management intervention among community-dwelling older adults and reports no RN-specific workload or staffing exposure. | The study was conducted in community/university outpatient settings rather than nursing homes or residential long-term care, and no RN-specific exposure-outcome relationship is reported. | Exclude |
| **23** | Konetzka, R. T., Stearns, S. C., & Park, J. (2008). The staffing–outcomes relationship in nursing homes. Health Services Research, 43(3), 1025–1042. https://doi.org/10.1111/j.1475-6773.2007.00803.x | Meets all core inclusion criteria. The study examines RN staffing as a distinct exposure (RN HPRD and RN skill mix) and analyses it separately from LPN and CNA staffing. | The setting is 1,366 freestanding nursing homes with 399,206 resident assessments; outcomes are resident pressure sores and urinary tract infections. Fixed-effects and instrumental-variable methods address endogeneity. | Include |
| 24 | Lee, T. W., Cho, S. Y., & Jang, Y. K. (2009). Predictors of Nursing Service Need for Nursing Homes Residents. Journal of Korean Academy of Nursing, 39(1), 95. https://doi.org/10.4040/jkan.2009.39.1.95 | The paper is published in Korean. Despite the presence of an English abstract, the full text including the introduction, methods, results, discussion, and conclusion is written entirely in Korean, failing the language inclusion criterion. | The study examines resident functional status as a predictor of nursing care time (framed as service need), rather than examining nurses' workload as an independent variable influencing resident care outcomes. The direction of inquiry is the inverse of the review's conceptual focus. | Exclude |
| 25 | Lingler, J. H., Sereika, S. M., Amspaugh, C. M., Arida, J. A., Happ, M. E., Houze, M. P., Kaufman, R. R., Knox, M. L., Tamres, L. K., Tang, F., & Erlen, J. A. (2016). An intervention to maximize medication management by caregivers of persons with memory loss: Intervention overview and two-month outcomes. Geriatric Nursing, 37(3), 186–191. https://doi.org/10.1016/j.gerinurse.2015.12.002 | The study evaluates a medication-management intervention for informal family caregivers of community-dwelling people with memory loss and reports no RN-specific workload or staffing exposure. | Intervention delivery and data collection occurred in participants' homes rather than nursing homes or residential long-term-care settings. | Exclude |
| 26 | Milstein, R., & Schreyoegg, J. (2020). The relationship between nurse staffing levels and nursing-sensitive outcomes in hospitals: Assessing heterogeneity among unit and outcome types. Health Policy, 124(10), 1056–1063. https://doi.org/10.1016/j.healthpol.2020.07.013 | The study is conducted exclusively in acute care hospitals across Germany, not in nursing homes or any long-term care setting. | The study is based on acute-care hospital units and therefore fails the review context criterion; it does not provide nursing-home RN-specific evidence. | Exclude |
| 27 | Mortuja, S. G., Dasgupta, S., Bhattacherjee, S., & Mukherjee, A. (2023). Nursing Personnel Workload and Staffing Need for Rural Hospitals of Darjeeling District, India: Assessment by Application of Workload Indicators of Staffing Need Tool (World Health Organization). Indian Journal of Public Health, 67(4), 654–659. https://doi.org/10.4103/ijph.ijph_1678_22 | The study was conducted in rural hospitals and primary health centres, not in nursing homes or residential long-term-care settings. | Although nursing workload is assessed using the WISN tool, the study addresses workforce planning and does not report an eligible resident outcome linked to an RN-specific nursing-home exposure. | Exclude |
| **28** | Mukamel, D. B., Saliba, D., Ladd, H., & Konetzka, R. T. (2022). Associations Between Daily Nurse Staffing Levels and Daily Hospitalizations and ED Visits in Nursing Homes. Journal of the American Medical Directors Association, 23(11), 1793–1799.e3. https://doi.org/10.1016/j.jamda.2022.06.030 | Daily RN hours are entered as a distinct exposure alongside LPN and CNA hours, permitting extraction of RN-specific coefficients. | The study includes 15,718 US nursing homes and resident hospitalisations/ED visits. Same-day and prior-day RN staffing estimates are reported separately; the resident service-use outcome therefore meets the revised criterion. | Include |
| 29 | Nguyen, T. T. H., Phung, H. T., & Bui, A. T. M. (2022). Applying the workload indicators of staffing needs method in nursing health workforce planning: Evidence from four hospitals in Vietnam. Human Resources for Health, 19(Suppl 1), 124. https://doi.org/10.1186/s12960-021-00668-y | The study was conducted in provincial general hospitals in Vietnam, not in nursing homes or residential long-term-care settings. | The WISN analysis addresses workforce planning and does not report an eligible resident outcome linked to an RN-specific nursing-home exposure. | Exclude |
| 30 | Ono, T., Nihei, M., Abiru, T., Higashibaba, K., & Kubota, T. (2023). Association between meaningful activities at home and subjective well-being in older adults with long-term care needs: A cross-sectional study. Geriatric Nursing, 52, 121–126. https://doi.org/10.1016/j.gerinurse.2023.05.013 | The study examines meaningful home activities and subjective wellbeing among older adults with long-term-care needs; no RN-specific workload or staffing exposure is analysed. | Data were collected through day-service facilities and home-visit nursing stations rather than residential nursing homes, and no eligible RN-specific exposure-outcome relationship is reported. | Exclude |
| 31 | Palmer, J. A., Parker, V. A., Berlowitz, D., Snow, A. L., & Hartmann, C. W. (2018). Resident Choice: A Nursing Home Staff Perspective on Tensions and Resolutions. Geriatric Nursing, 39(3), 271–278. https://doi.org/10.1016/j.gerinurse.2017.10.001 | The study examines staff perspectives on resident choice rather than an RN-specific workload or staffing exposure linked to an eligible resident outcome. | The sample includes multiple staff disciplines and nursing roles, and RN-specific findings are not reported separately. | Exclude |
| 32 | Toles, M., Leeman, J., McKay, M. H., Covington, J., & Hanson, L. C. (2022). Adapting the Connect-Home transitional care intervention for the unique needs of people with dementia and their caregivers: A feasibility study. Geriatric Nursing, 48, 197–202. https://doi.org/10.1016/j.gerinurse.2022.09.016 | The study evaluates a transitional-care intervention for people with dementia and their informal caregivers and reports no RN-specific workload or staffing exposure. | Regardless of facility type, the study does not examine an RN-specific workload or staffing exposure linked to an eligible resident outcome. | Exclude |

**Part B. Contextual evidence retained after RN-specific re-audit (n = 7)**

| **Citation** | **Why not eligible for RN-specific primary synthesis** | **Contextual data extracted** | **Use in revised manuscript** |
| --- | --- | --- | --- |
| Anderson, R. A., Issel, L. M., & McDaniel, R. R. Jr. (2003). Nursing Homes as Complex Adaptive Systems: Relationship Between Management Practice and Resident Outcomes. Nursing Research, 52(1), 12–21. https://doi.org/10.1097/00006199-200301000-00003 | Management practices (communication, decision-making, relationship-oriented leadership, formalisation) are the primary exposures; RN workload/staffing is not the exposure of interest. Resident outcomes are reported, but they cannot be attributed to a separable RN workload/staffing exposure. | Management practice and organisational context; resident-outcome information. | Context only: informs nursing-management and organisational interpretation; not used as RN-specific effect evidence. |
| Bowers, B. J., Lauring, C., & Jacobson, N. (2001). How nurses manage time and work in long-term care. Journal of Advanced Nursing, 33(4), 484–491. https://doi.org/10.1046/j.1365-2648.2001.01686.x | The qualitative sample comprises 18 licensed nurses; RN and LPN findings are not reported separately. The paper describes perceived consequences of time pressure for care but does not provide a separable RN-specific resident outcome. | Licensed-nurse time pressure, interruptions, task prioritisation, and work organisation. | Context only: informs workload conceptualisation; RN/LPN findings are not treated as separable RN evidence. |
| Chen, M., Goodwin, J. S., Bailey, J. E., Bowblis, J. R., Li, S., & Xu, H. (2023). Longitudinal associations of staff shortages and staff levels with health outcomes in nursing homes. Journal of the American Medical Directors Association, 24, 1755–1760.e7. https://doi.org/10.1016/j.jamda.2023.04.017 | The key shortage exposure combines aides and licensed nurses; a distinct RN-specific workload/staffing estimate is not available for the central analysis. Resident outcomes are reported, but the exposure fails the revised RN-separability criterion. | Mixed-staff shortages/staffing levels and resident health/functional outcomes. | Context only: informs staffing-shortage and organisational-capacity discussion; no RN-specific effect estimate used. |
| Munkeby, H., Bratberg, G., & Devik, S. A. (2023). Registered nurses’ exposure to high stress of conscience in long-term care. Nursing Ethics, 30, 1011–1024. https://doi.org/10.1177/09697330231167542 | The population includes RNs and the study provides RN-specific workforce information. The outcome is RN stress of conscience/workforce wellbeing rather than an eligible resident health, safety, service-use, medication-related, psychosocial, or quality-of-life outcome. | RN stress of conscience and workforce wellbeing. | Context only: informs workforce sustainability; no resident-outcome effect is attributed to RN workload. |
| Sinha, S., Mukamel, D. B., Saliba, D., Ladd, H., & Konetzka, R. T. (2023). New dimensions of staffing patterns in nursing homes and nursing home quality: Comparing staffing instability to staffing turnover. Journal of the American Medical Directors Association, 24(8), 1099–1105.e7. https://doi.org/10.1016/j.jamda.2023.04.009 | The central instability and turnover exposures are total nursing staffing measures rather than separable RN-specific exposures. Resident quality outcomes are reported, but RN-specific effects cannot be extracted for the primary exposure. | Total/mixed staffing instability, turnover, and nursing-home quality outcomes. | Context only: informs staffing-instability discussion; not used as separable RN effect evidence. |
| Tchouaket, E., Kilpatrick, K., & Jabbour, M. (2020). Effectiveness for introducing nurse practitioners in six long-term care facilities in Québec, Canada: A cost-savings analysis. Nursing Outlook, 68(5), 611–625. https://doi.org/10.1016/j.outlook.2020.06.002 | The intervention concerns nurse practitioners, not RN workload or RN staffing. Resident adverse events are reported, but the exposure is an NP model of care and therefore outside the RN-specific population/concept. | Nurse-practitioner model, adverse-event context, and cost outcomes. | Context only: informs advanced nursing/staffing-model discussion; outside RN-workload primary concept. |
| Väisänen, V., Ruotsalainen, S., Hietapakka, L., Sulander, J., & Sinervo, T. (2024). The role of workday characteristics on perceived stress and time pressure among nurses in Finnish long-term care: a cross-sectional study. BMC Health Services Research, 24, 878. https://doi.org/10.1186/s12913-024-11294-4 | The sample combines nursing personnel and does not provide a separable RN-specific resident-outcome estimate. Primary outcomes are nurses’ stress and time pressure, not resident outcomes. | Mixed-nursing-personnel workday characteristics, stress, time pressure, breaks, and task distribution. | Context only: informs experienced-workload interpretation; no separable RN resident-outcome effect used. |
